# CgMFS1, a Major Facilitator Superfamily Transporter, Is Required for Sugar Transport, Oxidative Stress Resistance, and Pathogenicity of *Colletotrichum gloeosporioides* from *Hevea brasiliensis*

**DOI:** 10.3390/cimb43030109

**Published:** 2021-10-11

**Authors:** Na Liu, Qiannan Wang, Chaozu He, Bang An

**Affiliations:** Hainan Key Laboratory for Sustainable Utilization of Tropical Bioresource, College of Tropical Crops, Hainan University, Haikou 570228, China; liuna202110@163.com (N.L.); wangqiannan@hainanu.edu.cn (Q.W.); czhe@hainanu.edu.cn (C.H.)

**Keywords:** *Colletotrichum gloeosporioides*, major facilitator superfamily, oxidative resistance, pathogenicity

## Abstract

*Colletotrichum gloeosporioides* is the main causal agent of anthracnose in various plant species. Determining the molecular mechanisms underlying the pathogenicity and fungicide resistance of *C. gloeosporioides* could help build new strategies for disease control. The major facilitator superfamily (MFS) has multiple roles in the transport of a diverse range of substrates. In the present study, an MFS protein CgMFS1 was characterized in *C. gloeosporioides.* This protein contains seven transmembrane domains, and its predicted 3D structure is highly similar to the reported hexose transporters. To investigate the biological functions of CgMFS1, the gene knock-out mutant ΔCgMFS1 was constructed. A colony growth assay showed that the mutant was remarkably decreased in vegetative growth in minimal medium supplemented with monosaccharides and oligosaccharides as the sole carbon sources, whereas it showed a similar growth rate and colony morphology as wild types when using soluble starch as the carbon source. A stress assay revealed that CgMFS1 is involved in oxidative stress but not in the fungicide resistance of *C. gloeosporioides*. Furthermore, its pathogenicity was significantly impaired in the mutant, although its appressorium formation was not affected. Our results demonstrate that CgMFS1 is required for sugar transport, resistance to oxidative stress, and the pathogenicity of *Colletotrichum gloeosporioides* from *Hevea brasiliensis.*

## 1. Introduction

The genus *Colletotrichum*, which comprises about 600 species, is one of the most common and notorious phytopathogens. Members of the genus attack a variety of crops throughout the world, which leads to heavy economic losses [1]. Most *Colletotrichum* species have a hemibiotrophic lifestyle, which comprises a biotrophic phase at the early infection stage and a necrotrophic phase later on [2]. Among these species, *Colletotrichum gloeosporioides* can cause anthracnose diseases in both aerial plant parts and in the post-harvest fruits of many important economic crops [3]. Genome and gene expression pattern analysis of *C. gloeosporioides* shows that the genes encoding the small secreted proteins and the secondary metabolite synthesis enzymes are upregulated at the biotrophic stage, whereas degradative enzymes are upregulated at necrotrophic stage [4]. *C. gloeosporioides* also causes anthracnose in *Hevea brasiliensis* in China and leads to great losses in rubber yield. Synthetic fungicides have been widely used to control anthracnose in *H. brasiliensis*; however, the indiscriminate use of synthetic fungicides can lead to pathogens becoming fungicide resistance and can cause environmental contamination. Thus, clarifying the molecular pathogenicity and fungicide resistance mechanisms of *C. gloeosporioides* might provide new clues for controlling anthracnose.

The major facilitator superfamily (MFS) is the largest group of secondary active membrane proteins and are found in all kingdoms of life [5]. These transporters mainly function in transporting a diverse range of substrates, including sugars, organic and inorganic ions, amino acids, and drugs [6,7]. At present, there are 105 distinct families of MFS that have been identified, with each family generally being responsible for the transport of a single class of compounds [5,8]. Some of these MFS families function as efflux pumps to extrude a variety of toxic materials out of cells and contribute to drug resistance and/or stress tolerance. The functions of yeast MFS in drug resistance have been well researched. In *Candida* *albicans,* the transporter FLU1 confers cell resistance to fluconazole, and CaMDR1 was identified because of its function of increasing resistance to benomyl and methotrexate in *Saccharomyces cerevisiae* [9,10]. Dtr1p from *S. cerevisiae* is not only involved in resistance to antimalarial drugs and organic acid preservatives, but also plays an essential role in spore wall formation [11]. In phytopathogenic fungi, prior research has mainly focused on the functions of MFS as multidrug transporters; moreover, some MFS transporters have also been found to participate in sugar transport and to contribute to fungal pathogenicity. In *Penicillium digitatum*, the causal agent of green mold disease in citrus, three MFS transporters, PdMfs1, Pdmfs2, and PdMFS1, are involved in the resistance to chemical fungicides and contribute to pathogenicity [12,13,14]. The *MFS1* gene of *Colletotrichum lindemuthianum* is only expressed at necrotrophic phase and is required for sugar utilization during interaction within the plant host [15]. In the *Colletotrichum fructicola*, the fungal pathogen of the tea oil camellia plant, the MFS transporter CfMfs1 has a role in sugar transport; in addition, the transporter also plays roles in conidiation and pathogenicity [16]. Bcmfs1 is involved in the protection of *Botrytis cinerea* against natural plant defense compounds during the pathogenic phase and antimicrobial compounds during its saprophytic growth phase [17]. In *Alternaria alternata*, AaMFS19 is required for resistance to oxidative stress and several kinds of fungicides, and it is required for full pathogenicity to the plant host; furthermore, the expression of *AaMFS19* is regulated by the Yap1 transcription activator, the Skn7 response regulator, and the MAP kinases [18]. However, the identification of MFS transporters is inadequate, and the functions of MFS proteins are still unknown in *C. gloeosporioides*.

In the present study, an MFS protein CgMFS1 was identified in *C. gloeosporioides* from *Hevea brasiliensis*. Through the construction the gene knock-out and complementation mutants, the functions of CgMFS1 in the regulation of growth, stress resistance, and pathogenicity of *C. gloeosporioides* were characterized.

## 2. Materials and Methods

### 2.1. Fungal Strains and Culture Conditions

*C. gloeosporioides* isolated from the *H. brasiliensis* was used as a wild type (WT) control. The genome sequence was uploaded to the NCBI database (BioSample: SAMN17266943 [https://www.ncbi.nlm.nih.gov/biosample/17266943, accessed on 9 January 2021]) in our previous work. All of the *C. gloeosporioides* strains were kept on potato dextrose agar (PDA) at 28 °C.

### 2.2. Bioinformatics Analysis

The homologues of CgMFS1 from other fungi were retrieved by searching against the NCBI GenBank database using BLASTP. Then, the bootstrap neighbor-joining phylogenetic tree was constructed using MEGA X under default settings and 1000 bootstrap replications [19]. The transmembrane domains of the protein were predicted with TMHMM Server v. 2.0 [20]. Protein homology modeling was performed with SWISS-MODEL [21].

### 2.3. Construction of Knock-Out and Complementation Mutants

A split-marker strategy was used for gene knock-out. First, the acetolactate synthase gene (*SUR*) cassette from *Magnaporthe oryzae* conferring resistance to chlorimuron ethyl (a sulfonylurea herbicide) was cloned into pBlueScript II SK (+) to construct pBSSUR; then, the 5′ and 3′ flanking fragments of CgMFS1 were amplified from genomic DNA using primer pairs the CgMFS1-5F/5R and CgMFS1-3F/3R and were cloned into pBSSUR to construct pBSSUR-CgMFS1-5′ and pBSSUR-CgMFS1-3′, respectively. After that, the flanking sequences together with the truncated resistant gene *SUR* were amplified with the primer pairs CgMFS1-5F/SURspl-R and SURspl-F/CgMFS1-3R, respectively. Protoplast preparation and transformation were performed as described in our previous work [22]. Then, the gene *CgMFS1* could be replaced by the two fragments using the homologous recombination strategy. To verify the correct integration of the recombinant fragments into the target locus, a two-round PCR diagnosis was conducted using primer pairs, with one primer being located outside of and one being located inside of the fragments (CgMFS1-SF/SUR-SR and SUR-SF/CgMFS1-SR). The homozygote of the correct transformants were further purified by single conidia isolations. Detection of the WT nuclei was conducted by the amplification of the CgMFS1 nucleotide sequence with the primer pair CgMFS1-OF/CgMFS1-OR. For the generation of the complementation mutants, the *CgMFS1* nucleotide sequence together with its native promoter (1.5 kb upstream) was amplified with CgMFS1-PF/CgMFS1-OR and was cloned into the plasmid with a trpC terminator (TtrpC) from *Aspergillus nidulans* and a hygromycin-resistant gene (HPT). Then, the linearized plasmid was transformed into the protoplasts of the CgMFS1 knock-out mutant, and a regeneration medium containing 300 μg mL^−1^ hygromycin was used to select the transformants. The positive transformants were further confirmed through the PCR diagnosis of CgMFS1 ORF. The primers that were used are listed in Supplementary Table S2.

### 2.4. Colony Growth Assay

Minimal medium (MM) (2 g L^−1^ NaNO_3_, 0.5 g L^−1^ KCl, 1 g L^−1^ KH_2_PO_4_, 0.5 g L^−1^ MgSO_4_·7H_2_O, 0.01 g L^−1^ FeSO_4_·7H_2_O, pH 6.9) supplemented with different sugars as the sole carbon sources were employed to analyze mycelium growth. In the present study, glucose, xylose, galactose, sucrose, maltose, and soluble starch, all at a 2% (*w*/*v*) concentration, were used. The WT and mutant strains were grown for 3 days in PDA medium, then disks of hypha with diameter of 4 mm were cut from the growing edge and were inoculated into the minimal medium. Fungal cultures were incubated at 28 °C, and the colony area was recorded. Each treatment contained three replicates, and all of the experiments were performed at least two times.

### 2.5. Stresses and Sensitivity Tests

To determine the sensitivity to stresses and fungicides, a colony growth assay was conducted. WT and mutant strains were grown for 3 days on PDA medium, then hypha disks with a diameter of 4 mm were cut from the growing edge and were inoculated on the minimal medium (with soluble starch as the sole carbon source) amended with 0.6 mol L^−1^ NaCl, 10 mmol L^−1^ H_2_O_2_, 0.5 mg mL^−1^ Congo red, 0.1 μg mL^−1^ Fludioxonil, 10 μg mL^−1^ Iprodione, 0.2 μg mL^−1^ Tebuconazole, and 2.5 μg mL^−1^ TriadiMefon. Fungal cultures were incubated at 28 °C, and the colony area was recorded. The relative inhibition ratio was determined by comparing the colony area with that of the CK, using the formula [(colony under stress/colony of CK) × 100%]. Each treatment contained three replicates, and all of the experiments were performed at least two times. All of the test chemicals were purchased from Sigma-Aldrich (St. Louis, MO, USA) or Sangon Biotech (Shanghai, China).

### 2.6. Quantitative RT-PCR Analysis

The wild-type strain was grown for 3 days on cellophane paper plated on the minimal medium supplemented with different chemicals, as mentioned above. Then, mycelium was collected and disrupted in liquid nitrogen by means of grinding in a mortar with a pestle, and the RNA was extracted using the RNAprep Pure Plant Plus Kit (TIANGEN Biotech, Beijing, China). Reverse transcription was conducted with FastKing gDNA Dispelling RT SuperMix (TIANGEN Biotech, Beijing, China) according to the manufacturer’s instructions. Quantitative RT-PCR analysis was performed with the QuantStudio 6 (Thermo Fisher, Waltham, MA, USA). The Actin coding gene was used as an endogenous control for normalization, and the relative expression levels were estimated using the 2^−ΔΔCt^ method. All of the reactions had three biological replicates.

### 2.7. Pathogenicity Assay

The pathogenicity assay was performed as described in our previous report [22]. Briefly, conidia were collected and resuspended in 0.5% malt extract broth (Difco) to a final concentration of 2 × 10^5^ conidia mL^−1^. The detached “light green” leaves from a rubber tree (Reyan 7-33-97) were pre-wounded with a sterile needle and were inoculated with droplets (5 μL) of the conidial suspensions. The inoculated leaves were kept in a moist chamber at 28 °C under natural illumination for 3 days, and the disease symptoms were scored. Each treatment contained three replicates of 10 leaves, and the entire experiment was repeated three times.

### 2.8. Appressorium Formation Assay

Onion epidermis was harvested and put on water agar plates. Conidia resuspended with ddH2O at a concentration of 2 × 10^5^ conidia mL^−1^ was used to inoculate the onion epidermis. After that, the inoculated epidermis was kept in a moist chamber at 28 °C for the desired time, and the infection structures were analyzed with a microscope. For the calculation of the appressorium rate, at least 50 conidia were counted under the microscope. Each treatment contained three replications, and the entire experiment was conducted twice.

### 2.9. Statistical Analysis

Data with a single variable were analyzed by one-way ANOVA, and mean separations were performed by Duncan’s multiple range test. Differences at *p* < 0.05 were considered significant.

## 3. Results

### 3.1. Identification of CgMFS1

**Figure 1:**
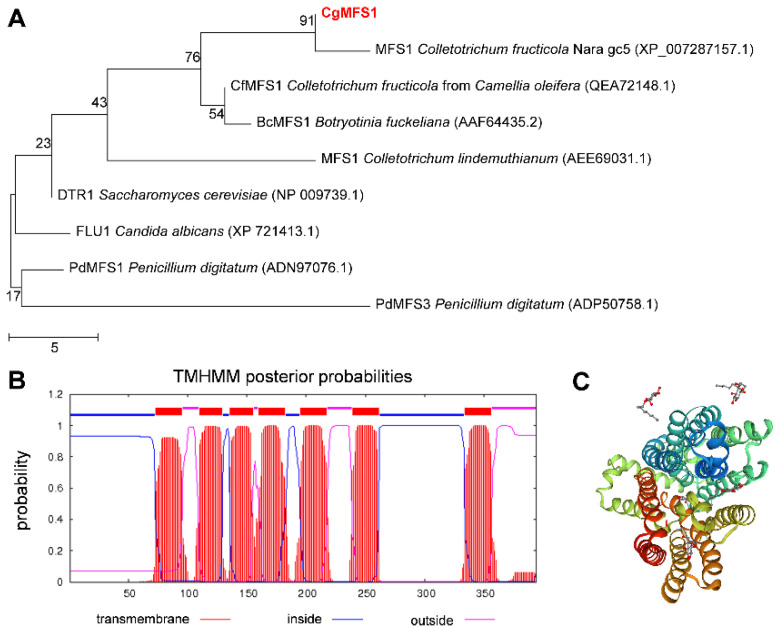
Bioinformatics analysis of CgMFS1. (**A**) The phylogenetic relationship of CgMFS1 and other fungal MFS proteins. (**B**) Predicted transmembrane domains of CgMFS1. (**C**) Predicted protein model of CgMFS1.

In the present study, a gene encoding an MFS protein was identified from the genome of *C. gloeosporioides,* and the gene was called CgMFS1. CgMFS1 was predicted to contain a 1185-bp open reading frame separated by four small introns, and it encodes a polypeptide of 394 amino acids (Supplementary Sequence Information). The CgMFS1 protein has a predicted molecular mass of 43.08 kDa and a predicted pI of 6.24. Phylogenetic tree analysis revealed that CgMFS1 is highly conserved in an amino acid sequence compared to some other reported fungal MFS transporters (Figure 1A). Transmembrane domains analysis revealed that CgMFS1 contains seven putative transmembrane domains (Figure 1B), and its predicted 3D structure was highly similar (with the coverage of 0.84) to the hexose transporter 1 of *plasmodium falciparum* (PfHT1) (Figure 1C).

### 3.2. Generation of Knock-Out and Complementation Mutants

**Figure 2:**
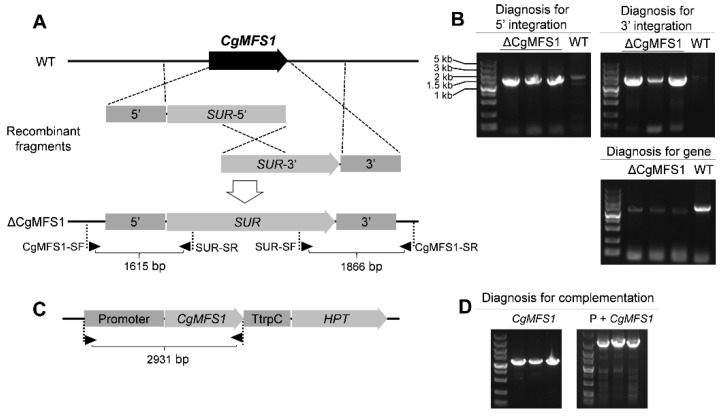
Strategies and diagnosis for the construction of *CgMFS1* knock-out and complementation mutants. (**A**) Split-marker strategy for *CgMFS1* knock-out. Diagnostic primers for integrations of the recombinant fragments are marked with black triangles. (**B**) Diagnosis for integrations of the recombinant fragments into the *CgMFS1* gene locus and the detection of *CgMFS1* nucleotide sequences in the knock-out mutants; ΔCgMFS1: the knock-out mutants; WT: wild type. (**C**) Strategy for *CgMFS1* complementation. (**D**) Diagnosis for *CgMFS1* nucleotide sequence, *CgMFS1,* and its native promoter (P + *CgMFS1*), respectively. P: promoter. The DNA marker DL5000 was used for the fragment sizes analysis.

To explore the biological functions of CgMFS1, the gene knock-out mutants were obtained using a split-marker strategy (Figure 2A). The PCR diagnosis showed that at least three transformants were identified with both upstream and downstream diagnostic fragments (Figure 2B), suggesting that the two recombinant fragments were correctly integrated into the *CgMFS1* locus. These transformants were further purified by single conidia isolation, and the following detection of the gene sequence showed that three mutants did not contain the *CgMFS1* nucleotide sequence in the genome, indicating that *CgMFS1* was completely knocked out from the genome of the mutants (Figure 2B). Then, the knock-out mutants were called ΔCgMFS1. Since the three randomly picked knock-out mutants showed similar phenotypes in terms of the growth rate and pathogenicity, only one strain was chosen for the construction of complementation mutants. The PCR diagnosis for the complementation mutants suggested that the CgMFS1-expression cassette was successfully integrated into the genome of the ΔCgMFS1 mutant (Figure 2C,D), and the mutants were called Res-ΔCgMFS1.

### 3.3. CgMFS1 Is Required for Carbon Utilization

**Figure 3:**
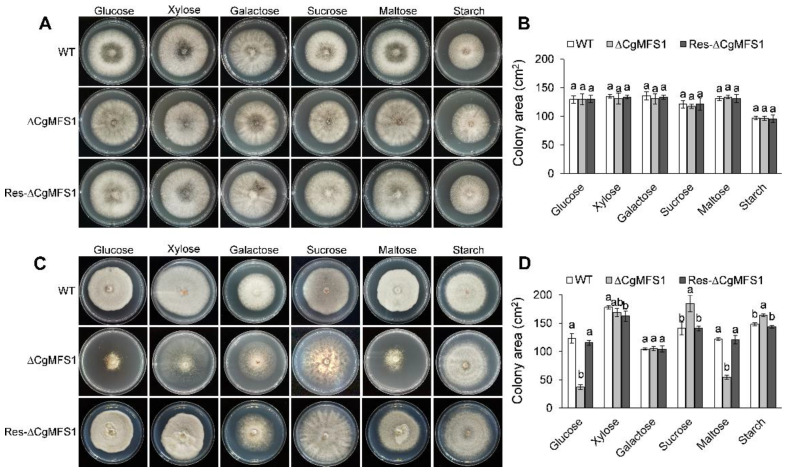
Colony morphology and area of *C. gloeosporioides* cultured in potato broth and minimal medium. (**A**) Colony morphology after growth in potato broth medium for 5 days. (**B**) Colony area after growth in potato broth medium for 5 days. (**C**) Colony morphology after growth in minimal medium for 7 days. (**D**) Colony area after growth in minimal broth medium for 7 days. Bars represent standard deviations (SD). Columns with different letters indicate significant difference (*p* < 0.05).

To determine the function of CgMFS1 in sugar transport, the *C. gloeosporioides* strains were incubated in potato broth and were minimally supplemented with medium different carbon sources. The colony growth assay showed that when incubated in the rich medium potato broth, there were no differences in the growth rate or in the colony morphology between the mutants and the wild types (WT) (Figure 3A,B). However, when cultured on the synthetic minimal medium with monosaccharides and oligosaccharides as the sole carbon sources, the ΔCgMFS1 colonies showed a significant decrease in the growth rate and/or the mycelium density compared to the WT and Res-ΔCgMFS1 (Figure 3C). For the mutant cultured on glucose and maltose, the colony area decreased by about 70% and 55%, respectively (Figure 3D). Meanwhile, when soluble starch was used as the carbon source, the ΔCgMFS1 colonies showed the same growth rate and similar colony morphology with the WT and the complementation mutant. Moreover, when incubated in liquid minimal medium, the biomass of the ΔCgMFS1 was significantly decreased when monosaccharides or oligosaccharides were used as the carbon sources (Supplementary Table S1). These results suggest that CgMFS1 is involved in the transport of monosaccharides and oligosaccharides but not starch.

### 3.4. CgMFS1 Is Required for Tolerance to Oxidative and Fungicide Stresses

**Figure 4:**
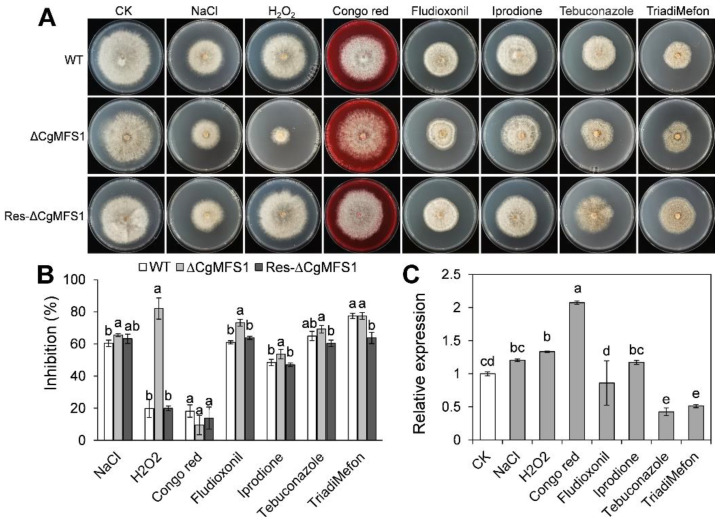
Colony growth and *CgMFS1* transcript levels under stresses. (**A**) Colony morphology after growth in minimal medium supplemented with different chemicals for 7 days. (**B**) Quantitative analysis of chemical sensitivity after growth for 7 days. The inhibition ratio was calculated by comparing the colony area with that of the CK. (**C**) Quantitative RT-PCR of transcript of *CgMFS1* under different chemical stresses. Bars represent standard deviations (SD). Columns with different letters indicate significant difference (*p* < 0.05).

According to the colony growth assay results, the minimal medium supplemented with soluble starch was used for the chemical sensitivity test. As shown in Figure 4A,B, the ΔCgMFS1 showed a remarkable increase in the sensitivity toward H_2_O_2_, with an inhibition ratio of up to 82%; in contrast, the inhibition rate of WT and Res-ΔCgMFS1 were both only 20%. Meanwhile, ΔCgMFS1 slightly increased the sensitivity to NaCl and the fungicides Fludioxonil, Iprodione, and Tebuconazole. Additionally, there was no difference in colony area between the mutants and the WT when put under Congo red and TriadiMefon stresses. To investigate whether these stresses influence the expression of *CgMFS1*, a qRT-PCR analysis was conducted. The results (Figure 4C) showed that the Congo red treatment showed a slight induction on *CgMFS1* transcription that was about 2-fold; and Tebuconazole and TriadiMefon down regulated the *CgMFS1* expression by about 0.5-fold.

### 3.5. CgMFS1 Plays a Role in Fungal Pathogenicity

**Figure 5:**
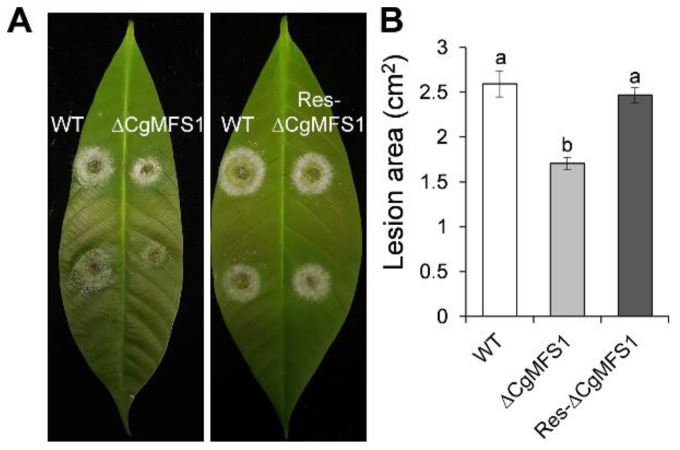
Pathogenicity assay. (**A**) Disease symptoms of rubber tree leaves at 3 days post inoculation. (**B**) Mean lesion area at 3 days post inoculation. Bars represent standard deviations (SD) of three groups. Columns with different letters indicate significant difference (*p* < 0.05).

Pathogenicity tests assayed on detached leaves showed that ΔCgMFS1 caused obviously smaller lesions than WT and Res-ΔCgMFS1 (Figure 5A,B), demonstrating ae lesion area of 1.71 cm^2^ compared to that of 2.59 cm^2^ for the WT and 2.47 cm^2^ for the Res-ΔCgMFS1.

### 3.6. CgMFS1 Is Not Required for Conidial Germination and Appressorium Formation

**Figure 6:**
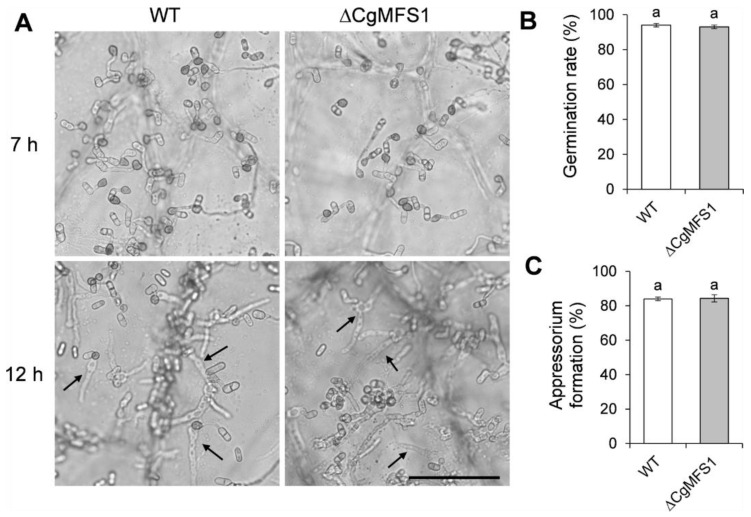
Conidia germination and appressorium formation on onion epidermis. (**A**) Microscopic view of conidia after incubation on onion epidermis for 7 and 12 h. The arrows indicate primary hyphae; Bar = 50 μM. (**B**) Conidia germination rate after incubation for 5 h. (**C**) Appressorium formation rate after incubation for 5 h. Bars represent standard deviations (SD) of three groups. Columns with different letters indicate significant difference (*p* < 0.05).

To further investigate whether CgMFS1 is required for appressorium formation, the conidial germination behavior was tested on onion epidermis. As shown in Figure 6, after incubation of 7 h, most of the ΔCgMFS1 conidia germinated and formed appressoria at a similar rate to that of the WT. Moreover, both the mutant and WT formed the primary hyphae and penetrated into the onion epidermis after being incubated for 12 h. The results suggest that CgMFS1 is not required for the conidial germination or appressorium formation of *C. gloeosporioides*.

## 4. Discussion

In the present study, a major facilitator superfamily protein CgMFS1 was identified in *C. gloeosporioides*. The alignment of the amino acid sequences showed that CgMFS1 has a high identity with other fungal MFS proteins, indicating that the MFS1 employs conserved functions in filamentous fungi (Figure 1). Protein structure prediction revealed that CgMFS1 is highly similar to the sole hexose transporter PfHT1 of *P. falciparum*, which belongs to the glucose transporter (GLUTs) family of the MFS families [23]. However, CgMFS1 only contains seven putative transmembrane domains; in comparison, the well-known GLUTs are all comprised of 12 transmembrane domains, such as human glucose transporters GLUTs and *Escherichia coli* xylose permease XylE [24,25], suggesting that there might be some differences in the biological functions between CgMFS1 and these GLUTs. Then, the function of CgMFS1 in sugar transport was investigated through the incubation of *C. gloeosporioides* on different carbon sources. It was found that the colony growth and biomass were remarkedly reduced in ΔCgMFS1 when monosaccharides or oligosaccharides were used as the sole carbon sources, but no difference was observed when starch was used as the carbon source. In addition, when incubated in the rich medium potato broth, the mutant showed no difference in terms of vegetative growth compared to WT and Res-ΔCgMFS1, regardless of the kind of carbon source that was used. These results suggest that CgMFS1 is involved in the transport of monosaccharides and oligosaccharides but not in the transport of starch in *C. gloeosporioides.*

Another important function of MFS proteins is to act as an efflux pump, and they also contribute to the drug resistance and/or the stress tolerance of cells [26]. For example, MFS-dependent efflux plays important roles in resistance to Fludioxonil, Iprodione, and Tebuconazole in *S. cerevisiae*, *B. cinerea*, and *A. alternate* [13,17,18]. During growth and interaction with plant hosts, phytopathogens are often challenged with oxidative stresses from their surroundings or from plant immunity reactions [27,28]. Thus, fungal pathogens have evolved multiple systems to eliminate excessive cellular reactive oxygen species (ROS), and one of the strategies is to pump H_2_O_2_ out of the cell via membrane transporters [29,30]. In the present study, the ΔCgMFS1 mutant displayed a significantly increased sensitivity to H_2_O_2_ stress and a slightly increased sensitivity to the fungicides Fludioxonil and Iprodione. The results suggest that that CgMFS1 is mainly involved in the oxidative stress response in *C. gloeosporioides*. To further investigate whether the transcription of *CgMFS1* is affected by these stresses, a qRT-PCR assay was conducted. The results showed that the expression of *CgMFS1* was not changed more than 2-fold when treated with these stresses, indicating that CgMFS1 has basal functions in *C. gloeosporioides*.

Pathogenicity tests showed that the ΔCgMFS1 mutant is reduced in its ability to cause necrotic lesions on detached *Hevea* leaves, revealing that CgMFS1 is required for full the pathogenicity of *C. gloeosporioides*. Conidia germination and appressorium formation are critical steps for *Colletotrichum* to infect the plant host [2]. In the present study, when inoculated on onion epidermis, the conidia of ΔCgMFS1 germinated and formed appressoria at the same rate as the WT, suggesting that the early infection process was not impaired in the mutant. After successful infection in the plant tissue, the pathogenic fungi would have been confronted with stresses resulting from host–plant immunity reactions, including oxidative burst, which could directly inhibit the pathogen growth or induce host hypersensitivity [22,31]. As shown in our stress assay, the sensitivity to H_2_O_2_ stress was significantly increased in ΔCgMFS1. Thus, we deduced that the reduction in the pathogenicity of ΔCgMFS1 is mainly due to its increased sensitivity to host-derived oxidative stress.

In summary, we have demonstrated CgMFS1 is required for sugar transport and pathogenicity and that it mediates resistance to oxidative stress in *C. gloeosporioides*. Our results lay a foundation for the further development of fungicides against *C. gloeosporioides*.

## Supplementary Materials

The supplementary files are available online at https://www.mdpi.com/article/10.3390/cimb43030109/s1.
