# Supplementary material for: CgMFS1, a Major Facilitator Superfamily Transporter, Is Required for Sugar Transport, Oxidative Stress Resistance, and Pathogenicity of Colletotrichum gloeosporioides from Hevea brasiliensis
Source: Curr Issues Mol Biol. 2021 Oct 11;43(3):1548–57. doi: 10.3390/cimb43030109 (PMC8929089; doi:10.3390/cimb43030109)
Supplement: Supplementary file 1 [file cimb-43-00109-s001.zip › cimb-1389191-supplementary.pdf]

Table S1. Biomass of *C. gloeosporioides* after culture in liquid minimal medium supplemented with difference carbon sources for 3 day. Different letters indicate significant difference (P < 0.05).

		**WT**	**ΔCgMFS1**	**Res-ΔCgMFS1**
**Glucose**	wet weight	0.274 ± 0.009**a**	0.025 ± 0.005**b**	0.266 ± 0.018**a**
	dry weight	0.062 ± 0.002**a**	0.007 ± 0.001**b**	0.063 ± 0.002**a**
**Xylose**	wet weight	0.378 ± 0.020**a**	0.140 ± 0.157**b**	0.370 ± 0.026**a**
	dry weight	0.094 ± 0.008**a**	0.033 ± 0.009**b**	0.089 ± 0.008**a**
**Galactose**	wet weight	0.228 ± 0.021**a**	0.122 ± 0.004**c**	0.196 ± 0.008**b**
	dry weight	0.055 ± 0.006**a**	0.022 ± 0.003**b**	0.047 ± 0.008**a**
**Sucrose**	wet weight	1.201 ± 0.106**a**	0.366 ± 0.043**b**	1.208 ± 0.046**a**
	dry weight	0.522 ± 0.044**a**	0.068 ± 0.003**c**	0.457 ± 0.031**b**
**Maltose**	wet weight	0.620 ±0.033**a**	0.071 ± 0.017**b**	0.601 ± 0.033**a**
	dry weight	0.139 ± 0.017**a**	0.028 ± 0.006**b**	0.150 ± 0.017**a**
**Starch**	wet weight	1.220 ± 0.083**a**	0.817 ± 0.023**b**	1.233 ± 0.072**a**
	dry weight	0.449 ± 0.013**a**	0.356 ± 0.017**b**	0.424 ± 0.035**a**

Table S2 Primers used in the present study.

primer	sequence	Restriction
CgMFS1-5F	gcggccgcGCTGGTGAGACATTGAA	Not I
CgMFS1-5R	ggatccGATGCTTTCTGAACTCTT	BamH I
CgMFS1-3F	gaattcATGGTATCAAACAAGTGG	EcoR I
CgMFS1-3R	gggcccAAGACCGTGAACAAACA	Apa I
SUR-SPLF	CCTCTGATATTGGAAGCGACGC	–
SUR-SPLR	ATGTTGGCATAAGCCGAACCGT	–
CgMFS1-SF	CCCATCTCGTTTCGCCACTTT	–
SUR-SR	GCGTTTGTAACTCTGCCTGTTTG	–

SUR-SF	ACGAGGACCGCTACTCACATAC	–
CgMFS1-SR	CCAAGACTCAAGAAGCCAAGAC	
CgMFS1-OF	tctagaATGAACACTGTCCCTCAG	Xba I
CgMFS1-OR	ggatccAGTGCTCAGAATAGGCCC	BamH I
CgMFS1-PF	ctgcagCTGCAGACGAAGCGCCGTTGGGAATG	Pst I
QCgMFS1-F	CTGGGCGGCTATTAACGTATG	–
QCgMFS1-R	GTCCCCAAACACACGCAAGT	–
Qcgactin-F	ATCCAGGCCGTCCTGTCTCT	–
Qcgactin-R	CAAGTCACGACCAGCCATGT	–

Sequence information

>*CgMFS1* nucleotide sequence

ATGAACACTGTCCCTCAGCGTGGGAGCGTTGACGATGACCCTGTGGAAAA
GGGGGATGATCTCGGAAAGCCGATTACGCCCATCCAGAGCCATGGCCGTG
GCACAACGGTCGAATACACATCCGACAATACACATGCACTCAGTGAAGTG
GAAATAAACCTCAGGGTGATTGACGAAGCTGTCGAGGCCATTGGATTCGG
AAAGTTCCAATGGCAGCTGGCCCTTTCCTGTGGATTTGGCTTCCTCGCGGA
TCAGGTCGGTACCCAGGCATTCGTCAAATGCAGACGCTAACACGGACGTA
AATGCAGATGCTCCTCGTGTCCATCTCTCTCGTCGGACCCCAGCTCATTCC
CGAGTTCGCGCCAAAACACTCCACTTTGCTCCCGGCGTCCAACTACGCTG
GCTTGCTCATCGGCGCGGTTTTGATGGGCCTGCTTGCGGATAATATTGGGA
GGAGGATGGTGTGGCAGCTGTCGATATTCGGTATCTCTATTGCTACTATGC
TGGCTGCTTCATCTCCTAACTGGGCGGCTATTAACGTATGGGTTGCTATTT
GTGGCTTCTTCGGAGGCGGAAACCGTAAGTCTCTCTACAGGGGACTAGGA
AATGACTGATGCTGACAACACTGTTGCTCCCCACAGTCGCGATTGATCTTA
CAATTCTTGCCGAGAATATCCCTCAGCGATGGTCTTTTATGCTTGCTGGAC
TTGCGTGTGTTTGGGGACTAGGGAATACTATCACCGGTATCTTCGGTAAGA
CTCGCTGATCATCCCTACTTACATAGGGTCAAATTGGCACAAAAGCTGAA
TCCTAGTCTAGGATGGGCCCTTATTGTCCCCTTCAGTTGCCCACAGGATGC
AACACCAGAGACCTGTCCCAAGTCCGCAAACATGGGTTGGCGGTATCTGT
ACATCCTCCTGGGTGGGTTATGCCTTGTAATGTCAATTATTCGGGCTCTAG
TACTTCGGACACACGAGTCTCCCAGATGGCTAGTGACTTGCGGCCGCATC
GACGAGGGCGTGGATGTCATCAACCGTATTAGCGCGATGAACAGGTCGAG
CTATACGATTTCTGCCGATCAGTTCATCAGAATTGGGTCCACAGAAGAAG
TGAAGACGATGTCATTTGGCGAAAACATTCACCGAGCGAGCAGGTTATTC
AAGGGAAAGACACAAATCAGATTGATGATCTGTCTGACGATGCTGTGGAT
GTTGGTTGGTATTGCGTAAGTCTTCGCTATCATCTCATACAGCGTTGAGAA
AGTCGAATGACTAATTTCTCAACTTCAGGTACCCTCTTTTTACAATCTTCTT
GCCCTACTATCTGCGCGCACACGGCGCGGACCTTGGCGACTCAAGTACCT

ACACCACATACCGAGATTGGACAATCTCGTCAGTCGTTGGCACATTCGGG
CCTATTCTGAGCACTTGA

>*CgMFS1* ORF

ATGAACACTGTCCCTCAGCGTGGGAGCGTTGACGATGACCCTGTGGAAAA
GGGGGATGATCTCGGAAAGCCGATTACGCCCATCCAGAGCCATGGCCGTG
GCACAACGGTCGAATACACATCCGACAATACACATGCACTCAGTGAAGTG
GAAATAAACCTCAGGGTGATTGACGAAGCTGTCGAGGCCATTGGATTCGG
AAAGTTCCAATGGCAGCTGGCCCTTTCCTGTGGATTTGGCTTCCTCGCGGA
TCAGATGCTCCTCGTGTCCATCTCTCTCGTCGGACCCCAGCTCATTCCCGA
GTTCGCGCCAAAACACTCCACTTTGCTCCCGGCGTCCAACTACGCTGGCTT
GCTCATCGGCGCGGTTTTGATGGGCCTGCTTGCGGATAATATTGGGAGGA
GGATGGTGTGGCAGCTGTCGATATTCGGTATCTCTATTGCTACTATGCTGG
CTGCTTCATCTCCTAACTGGGCGGCTATTAACGTATGGGTTGCTATTTGTG
GCTTCTTCGGAGGCGGAAACCTCGCGATTGATCTTACAATTCTTGCCGAGA
ATATCCCTCAGCGATGGTCTTTTATGCTTGCTGGACTTGCGTGTGTTTGGG
GACTAGGGAATACTATCACCGGTATCTTCGGATGGGCCCTTATTGTCCCCT
TCAGTTGCCCACAGGATGCAACACCAGAGACCTGTCCCAAGTCCGCAAAC
ATGGGTTGGCGGTATCTGTACATCCTCCTGGGTGGGTTATGCCTTGTAATG
TCAATTATTCGGGCTCTAGTACTTCGGACACACGAGTCTCCCAGATGGCTA
GTGACTTGCGGCCGCATCGACGAGGGCGTGGATGTCATCAACCGTATTAG
CGCGATGAACAGGTCGAGCTATACGATTTCTGCCGATCAGTTCATCAGAA
TTGGGTCCACAGAAGAAGTGAAGACGATGTCATTTGGCGAAAACATTCAC
CGAGCGAGCAGGTTATTCAAGGGAAAGACACAAATCAGATTGATGATCTG
TCTGACGATGCTGTGGATGTTGGTTGGTATTGCGTACCCTCTTTTTACAAT
CTTCTTGCCCTACTATCTGCGCGCACACGGCGCGGACCTTGGCGACTCAAG
TACCTACACCACATACCGAGATTGGACAATCTCGTCAGTCGTTGGCACATT
CGGGCCTATTCTGAGCACTTGA

>CgMFS1 amino acid sequence

MNTVPQRGSVDDDPVEKGDDLGKPITPIQSHGRGTTVEYTSDNTHALSEVEIN
LRVIDEAVEAIGFGKFQWQLALSCGFGFLADQMLLVSISLVGPQLIPEFAPKHS
TLLPASNYAGLLIGAVLMGLLADNIGRRMVWQLSIFGISIATMLAASSPNWA
AINVWVAICGFFGGGNLAIDLTILAENIPQRWSFMLAGLACVWGLGNTITGIF
GWALIVPFSCPQDATPETCPKSANMGWRYLYILLGGLCLVMSIIRALVLRTHE
SPRWLVTCGRIDEGVDVINRISAMNRSSYTISADQFIRIGSTEEVKTMSFGENIH
RASRLFKGKTQIRLMICLTMLWMLVGIAYPLFTIFLPYYLRAHGADLGDSSTY
TTYRDWTISSVVGTFGPILST
